# Exact traveling wave solutions of modified KdV–Zakharov–Kuznetsov equation and viscous Burgers equation

**DOI:** 10.1186/2193-1801-3-105

**Published:** 2014-02-21

**Authors:** Md Hamidul Islam, Kamruzzaman Khan, M Ali Akbar, Md Abdus Salam

**Affiliations:** Department of Electronics and Telecommunication Engineering, Prime University, Dhaka, 1216 Bangladesh; School of Biomolecular and Physical Sciences, Griffith University, Kragujevac, Australia; Department of Mathematics, Pabna University of Science and Technology, Pabna, 6600 Bangladesh; Department of Applied Mathematics, University of Rajshahi, Rajshahi, 6205 Bangladesh; Department of Mathematics, Mawlana Bhashani Science and Technology University, Tangail, 1902 Bangladesh

**Keywords:** Enhanced (*G '/G*)-expansion method, Modified KDV-ZK equation, Viscous burgers equation, Traveling wave, Solitary wave

## Abstract

**Abstract:**

Mathematical modeling of many physical systems leads to nonlinear evolution equations because most physical systems are inherently nonlinear in nature. The investigation of traveling wave solutions of nonlinear partial differential equations (NPDEs) plays a significant role in the study of nonlinear physical phenomena. In this article, we construct the traveling wave solutions of modified KDV-ZK equation and viscous Burgers equation by using an enhanced (*G '/G*) -expansion method. A number of traveling wave solutions in terms of unknown parameters are obtained. Derived traveling wave solutions exhibit solitary waves when special values are given to its unknown parameters.

**Mathematics subject classification:**

35C07; 35C08; 35P99

## Background

Engineers, physicists, and mathematicians have always shown their incessant interest in studying nonlinear problems related to numerous scientific applications, such as fluid dynamics, high-energy physics, plasma physics, elastic media, optical fibers, biomathematics, chemical kinematics, chemical physics and geochemistry. Many young scientists have also shown their increased interest in last two decades because of plausible development of nonlinear science during this period of time. In order to understand the behavior of a nonlinear phenomenon we need to solve the nonlinear equation/set of equations describing that phenomenon, is often very much challenging. There are so many approaches developed over years to analyze/solve such system of nonlinear equations, most of them are based on some assumptions, and hence approximations. Though perturbation methods, like other nonlinear analysis techniques, have their own limitations, are most useful methods among all these approaches so far. Using perturbation method, to achieve the ideal results an appropriate choice of small parameter has to be made efficiently, otherwise, a fatal error in results may occur. The perturbation methods are not even applicable to many nonlinear equations because of not having small parameter, which is the principal assumption that has to be met by an equation in order to apply perturbation method. Furthermore, the approximate solutions obtained using perturbation methods are valid only for the small values of the parameters (Ghorbani and Saberi-Nadjafi [2007]; Mohiud-Din [2007]; Mohyud-Din and Noor, [2009]). The investigation of exact traveling wave solutions to these nonlinear equations (NPDEs) have also been observed as a field of great interest to many mathematicians and physicists because of its significant role in understanding the behavior of nonlinear physical phenomena. As a result, numerous techniques of obtaining traveling wave solutions have been developed over last three decades, such as, the Hirota’s bilinear transformation method (Hirota [1973, 1981]), the modified simple equation method (Jawad et al. [2010]; Khan and Akbar [2013a]; Ahmed et al. [2013]; Zayed and Hoda [2013]; Zayed and Arnous [2012]), the tanh-function method (Wazwaz [2005]; Parkes and Duffy [1996]), the Exp-function method (He and Wu [2006]; Akbar and Ali [2011b]; Bekir and Boz [2008]; Xu et al. [2009]), the Jacobi elliptic function method (Ali [2011]), the (*G '/G*) -expansion method (Akbar et al. [2012a, 2012b]; Akbar and Ali [2011a]; Wang et al. [2008]; Shehata [2010]; Koll and Tabi [2011]; Naher et al. [2013]; Zayed [2009, 2010]; Aslan [2010]; Bekir and Aksoy [2012]), the homotopy perturbation method (Mohiud-Din [2007]; Mohyud-Din and Noor [2009]), transformed rational function method (Ma and Jyh [2009]; Ma and Fuchssteiner [1996]), multiple exp-function method (Ma et al. [2010]; Ma and Zhu [2012]), generalize Hirota bilinear method (Ma [2013]), enhanced (*G '/G*) -expansion method (Khan and Akbar [2013b]), The Sine-Cosine method (Bibi and Mohyud-Din [2013]), the first integral method (Tascan and Bekir [2010]; Feng [2002]), the ansatz method (Hu [2001a, 2001b]) and many others.

The present article is devoted to construct the exact solutions for modified KDV-ZK equation and viscous Burgers equation using a relatively new technique, named, enhanced (*G '/G*) -Expansion method. The rest of the paper is organized as follows. Details explanation of enhanced (*G '/G*) -expansion method has been presented in the next section. The obtained solutions of modified KDV-ZK equation and viscous Burgers equation using this method are presented in Section 3. In Section 4, we have presented some graphs of obtained family of solutions for some particular values of the unknown parameters and the final conclusions are shown in Section 5.

### Enhanced (*G '/G*) -expansion method

In this section we describe in details the enhanced (*G '/G*) -expansion method for finding traveling wave solutions of nonlinear evolution equations. Any nonlinear evolution equation in two independent variables *x* and *t* can be expressed in following form:2.1

where *u*(*ξ*) = *u*(*x*, *t*) is an unknown function, *R* is a polynomial of *u*(*x*, *t*) and its partial derivatives in which the highest order derivatives and nonlinear terms are involved. The following steps are involved in finding the solution of nonlinear Equation (2.1) using this method (Khan and Akbar [2013b]):

Step 1: The given PDE (2.1) can be transformed into ODE using the transformation *ξ* = *x* ± *Wt*, where *W* is the speed of traveling wave such that *W* ∈ *R* - {0}.

The traveling wave transformation permits us to reduce Eq. (2.1) to the following ODE:2.2

where *R* is a polynomial in *u*(ξ) and its derivatives, where , and so on.

Step 2: We now suppose that Eq. (2.2) has a general solution of the form2.3

subject to the condition that *G* = *G*(*ξ*) satisfy the equation2.4

where *a*_*i*_, *b*_*i*_(-*n* ≤ *i* ≤ *n*; *n* ∈ *N*) and *λ* are constants to be determined, provided that *σ* = ± 1 and *μ* ≠ 0.

Step 3: The positive integer *n* can be determined by balancing the highest order derivatives to the highest order nonlinear terms appear in Eq. (2.1) or in Eq. (2.2). More precisely, we define the degree of *u*(*ξ*) as *D*(*u*(*ξ*)) = *n* which gives rise to the degree of other expression as follows:2.5

Step 4: We substitute Eq. (2.3) into Eq. (2.2) and use Eq. (2.4). We then collect all the coefficients of (*G '/G*)^*j*^ and  together. Since Eq. (2.3) is a solution of Eq. (2.2), we can set each of the coefficient equal to zero which leads to a system of algebraic equations in terms of *a*_*i*_, *b*_*i*_(-*n* ≤ *i* ≤ *n*; *n* ∈ *N*), *λ*, and *W*. One can solves easily these system equations using Maple.

Step 5: For *μ* < 0 general solution of Eq. (2.4) gives2.6

and2.7

And for *μ* > 0, we get2.8

and2.9

where *A* is an arbitrary constant. Finally, we can construct a number of families of traveling wave solutions of Eq. (2.1) by substituting the values of *a*_*i*_, *b*_*i*_(-*n* ≤ *i* ≤ *n*; *n* ∈ *N*), *λ*, and *W* (obtained in Step 3) and using Eqs. (2.6) - (2.9) into Eq. (2.3).

### Applications of enhanced (*G '/G*) -expansion method to modified KDV-ZK equation and viscous Burgers equation

#### Modified KDV-ZK equation

In this current sub-section, we apply enhanced (*G '/G*) -expansion method to solve the modified KDV-ZK equation of the form,3.1

where d is a nonzero constant.

The traveling wave transformation equation *u*(*ξ*) = *u*(*x*, *t*), *ξ* = *x* + *y* + *z* - *Wt* transform Eq. (3.1) into the following ordinary differential equation:3.2

Integrating Eq. (3.2) with respect to *ξ*, we obtain3.3

where *R* is a constant of integration. Following the process as described in step 3 (Section 2), balance between the highest-ordered derivative term *u*'' and nonlinear term *u*^3^ of Eq. (3.3) provides *n* = 1.

For *n* = 1 Eq. (2.3) takes the following form:3.4

where *G* = *G*(*ξ*) satisfies Eq. (2.4). Substituting Eq. (3.4) into Eq. (3.3) and using Eq. (2.4), we get a polynomial in (*G* '/*G*)^*j*^ and . Setting the coefficient of (*G* '/*G*)^*j*^ and  equal to zero, we obtain a system containing a large number of algebraic equations in terms of unknown coefficients. We have solved this system of equations using Maple 13 and obtained the following set of solutions:

Set 1: .

Set 2: .

Set 3: .

Set 4: .

Set 5: .

Set 6: .

Corresponding to each set of solution we get two different families of traveling wave solutions of Eq. (3.1) according as *μ <* 0 and *μ >* 0. In doing so, we have obtained twelve families of traveling wave solutions while each family comprises of two solutions.

First we represent the families of hyperbolic solutions corresponding to *μ <* 0:

Family 1:

where *ξ* = *x* + *y* + *z* + 12*μt*.

Family 2:

where *ξ* = *x* + *y* + *z* - 6*μt*.

Family 3:

where .

Family 4:

where *ξ* = *x* + *y* + *z* - 6*μt*.

Family 5:

where *ξ* = *x* + *y* + *z* + 3*μt*.

Family 6:

where *ξ* = *x* + *y* + *z* + 3*μt*.

And, the families of plane periodic solutions corresponding to *μ > 0* are:

Family 7:

where *ξ* = *x* + *y* + *z* + 12*μt*.

Family 8:

where *ξ* = *x* + *y* + *z* - 6*μt*.

Family 9:

where .

Family 10:

where *ξ* = *x* + *y* + *z* - 6*μt*.

Family 11:

where *ξ* = *x* + *y* + *z* + 3*μt*.

Family 12:

where *ξ* = *x* + *y* + *z* + 3*μt*.

**Remark 1**: All the obtained solutions have been checked with Maple by putting them back into the original equations and found correct.

#### Viscous Burgers equation

In this sub-section, we will apply enhanced (*G '/G*) -expansion method to solve the viscous Burgers equation of the form,3.5

where *v* is the viscosity coefficient. Burgers equation is a model for nonlinear wave propagation, especially in fluid mechanics. It occurs in various areas of applied mathematics, such as modeling of gas dynamics and traffic flow.

The traveling wave transformation equation *u*(*ξ*) = *u*(*x*, *t*), *ξ* = *x* - *Wt* transform Eq. (3.5) into the following ordinary differential equation:3.6

Integrating Eq. (3.6) with respect to *ξ* setting constant of integration to zero, we obtain3.7

Following the process as described in step 3 (Section 2), balance between the highest-ordered derivative term *u*' and nonlinear term *u*^2^ of Eq. (3.7) provides *n =* 1.

For *n =* 1 Eq. (2.3) takes the following form:3.8

where *G* = *G*(*ξ*) satisfies Eq.(2.4). Substituting Eq. (3.8) into Eq. (3.7) and using Eq. (2.4), we get a polynomial in (*G '/G*)^j^ and . Setting the coefficient of (*G* '/*G*)^*j*^ and  equal to zero, we obtain a system containing a large number of algebraic equations in terms of unknown coefficients. We have solved this system of equations using Maple 13 and obtained the following set of solutions:

Set 1: .

Set 2: ,

Set 3: .

Set 4: .

Set 5: .

Set 6: .

According to the parallel course of action the families of hyperbolic solutions corresponding to *μ < 0* are:

Family 1:

where .

Family 2:

where .

Family 3:

where .

Family 4:

where .

Family 5:

where .

Family 6:

where .

Similarly, we can write down the other families of exact trigonometric function solutions corresponding to *μ >* 0 which are omitted for convenience.

**Remark 2**: All the obtained solutions have been checked with Maple by putting them back into the original equations and found correct.

### Graphical representation of solitary waves (obtained from above family of traveling waves)

#### Modified KDV-ZK Equation

We have obtained total twenty four solution profiles of traveling waves in terms of some unknown parameters; subdivided (above) into twelve families according as negative or positive values of *μ*. All of these solutions are combination of hyperbolic functions or trigonometric functions but Family-4 and Family-10 which are combination of algebraic functions, and hyperbolic functions or trigonometric functions. Solitary waves can be obtained from each traveling wave solution by setting particular values to its unknown parameters. If we put *a*_0_ = 0, then Family 4 and Family 10 coincide with Family 2 and Family 8 respectively. In this section, we have presented some graphs of solitary waves constructed by taking suitable values of involved unknown parameters.

**Figure 1:**
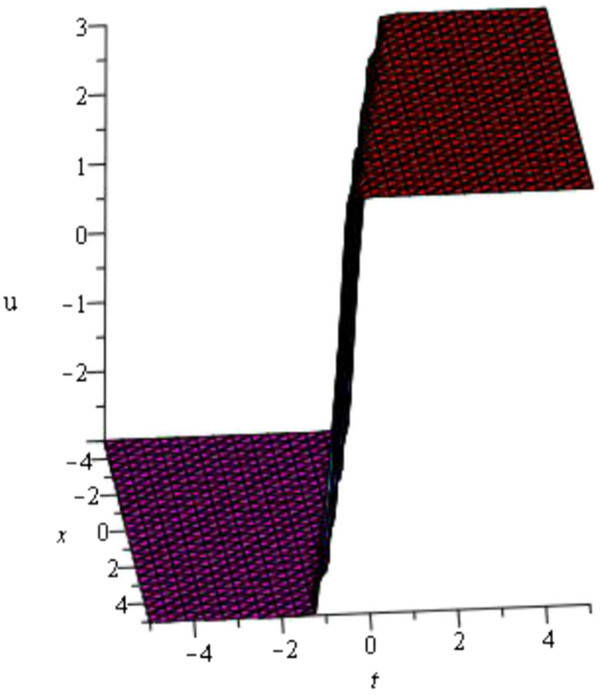
**Kink wave profile of mKDV-ZK equation.**

**Figure 2:**
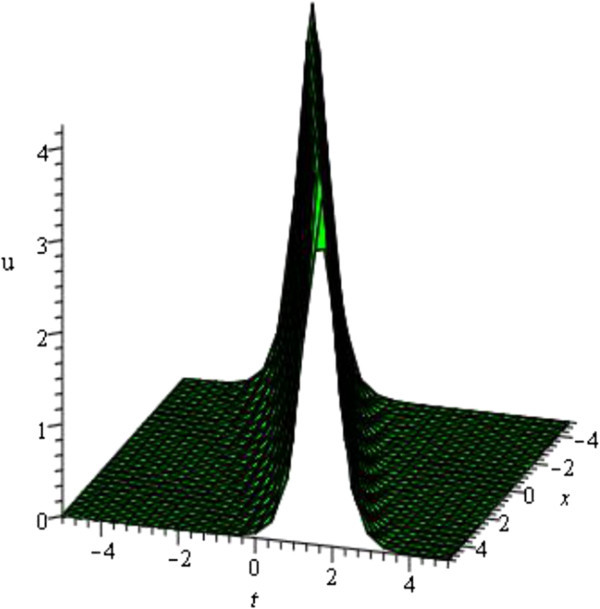
**Soliton profile of mKDV-ZK equation.**

**Figure 3:**
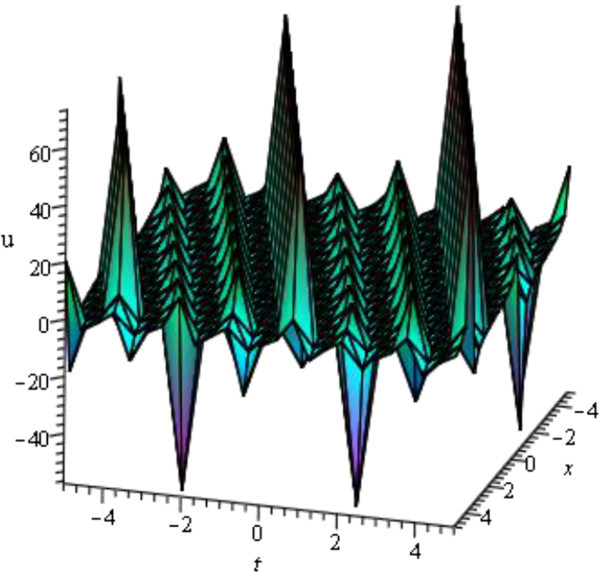
**Periodic wave profile of mKDV-ZK equation.**

**Figure 4:**
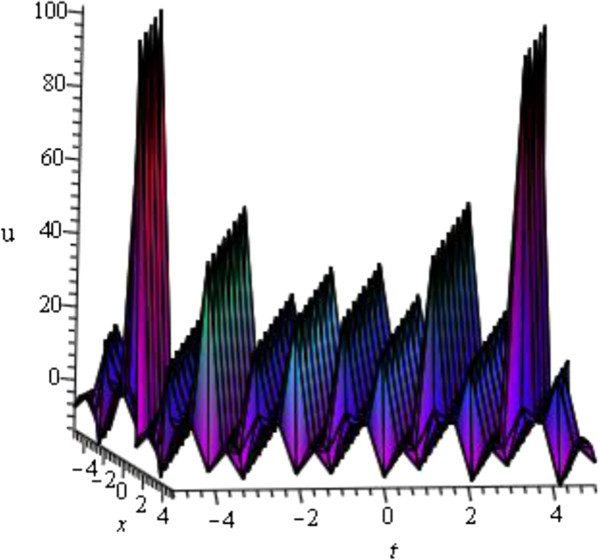
**Periodic wave profile of mKDV-ZK equation.**

- For the values of *μ* = - 1, *d* = - 2, *A* = *y* = *z* = 0 within the interval - 5 ≤ *x*, *t* ≤ 5 Figure 1 is kink wave (only shows the shape of *u*_3_(*ξ*) of mKDV-ZK equation).

- For the values of *μ* = - 1, *d* = 1, *A* = *y* = *z* = 0 within the interval - 5 ≤ *x*, *t* ≤ 5 Figure 2 is soliton wave (only shows the shape of *u*_12_(*ξ*) of mKDV-ZK equation).

- For the values of *μ* = 0.50, *d* = - 1, *A* = 3, *y* = *z* = 0 within the interval - 5 ≤ *x*, *t* ≤ 5, Figure 3 is a periodic wave (only shows the shape of *u*_15_(*ξ*) of mKDV-ZK equation).

- For the values of *μ* = 1, *d* = - 2, *A* = *y* = *z* = 0 within the interval - 5 ≤ *x*, *t* ≤ 5, *u*_24_(*ξ*) (solution of mKDV-ZK equation) shows the shape of periodic wave represented in Figure 4.

#### Viscous Burgers equation

Now we will discuss some of the obtained results of viscous Burgers equation and their graphical representations.

**Figure 5:**
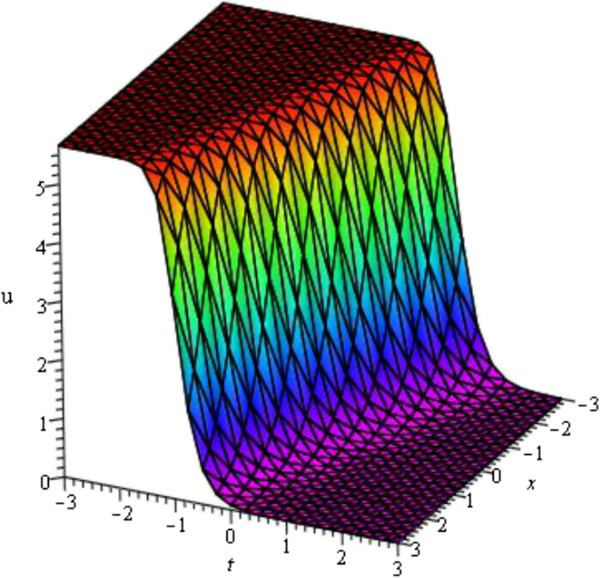
**Kink profile of viscous Burgers equation (Shape of** ***u***_**1**_ **(** ***ξ*** **)).**

**Figure 6:**
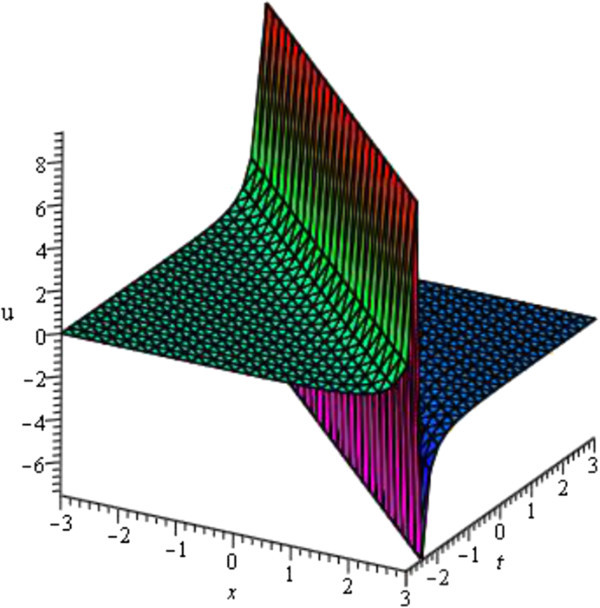
**Singular kink profile of viscous Burgers equation (Shape of** ***u***_**3**_ **(** ***ξ*** **)).**

For the values of *A* = 0, *μ* = - 2, *σ* = 1 and *ν* = - 2 within the interval - 3 ≤ *x*, *t* ≤ 3 the solution of viscous Burgers equation *u*_1_(*ξ*) shows Kink wave which is represented in Figure 5 and For the values of *a*_0_ = 0, *A* = 0, *μ* = - 1, *σ* = - 1 and *ν* = - 0.50 within the interval - 3 ≤ *x*, *t* ≤ 3 the solution of viscous Burgers equation *u*_3_(*ξ*) shows singular kink wave which is represented in Figure 6. Some graphical representations of viscous Burgers equation are given below:

## Conclusions

In this article, we obtain a large number of exact traveling wave solutions including solitary wave solutions for modified KDV-ZK equation and viscous Burgers equation through enhanced (*G '*/*G*))-expansion method. Using this method, we have found some extra family of solutions, is the best thing about this method. The obtained solutions suggest that enhanced (*G '*/*G*))-expansion method can be used as useful mathematical tool for solving nonlinear evolution equations arises in the arena of mathematical physics, engineering sciences and applied mathematics.
